# Two-step hot isostatic pressing densification achieved non-porous fully-densified wood with enhanced physical and mechanical properties

**DOI:** 10.1038/s41598-023-41342-8

**Published:** 2023-08-31

**Authors:** J. C. Maturana, P. Guindos, J. Lagos, C. Arroyave, F. Echeverría, E. Correa

**Affiliations:** 1 Grupo de Investigación Materiales con Impacto – MAT&MPAC, Facultad de Ingenierías, Universidad de Medellín UdeMedellín, Carrera 87 No. 30 – 65, Medellín, 050026 Colombia; 2 Centro Nacional de Excelencia Para la Industria de la Madera (CENAMAD), School of Engineering, Pontificia Universidad Católica de Chile, Vicuña Mackenna 4860, Santiago, Chile; 3 Grupo de Investigaciones y Mediciones Ambientales – GEMA, Department of Environmental Engineering, Universidad de Medellín UdeMedellín, Carrera 87 No. 30 – 65, Medellín, 050026 Colombia; 4 Centro de Investigación, Innovación y Desarrollo de Materiales – CIDEMAT, Facultad de Ingeniería, Universidad de Antioquia UdeA, Calle 70 No. 52-21, Medellín, Colombia; 5 Grupo de Investigación Valoración y Aprovechamiento de la Biodiversidad - VALORABIO, Universidad Tecnológica del Chocó UTCH, Carrera 22 No. 18B – 10, Quibdó, Colombia

**Keywords:** Materials science, Structural materials

## Abstract

A new two-step densification method for wooden materials entitled hot isostatic pressing (HIP) is proposed. This method has the advantage over previous densification methods that can achieved almost the full densification of wood, reaching values up to 1.47 kg/m^3^, which exceeds any value ever reported for a hardwood species. Furthermore, it can preserve about 35% of the original volume, in comparison to other methods which typically can preserve only 20% of the volume. Although not tested in this investigation, in principle, the HIP method should be capable of densifying any shape of wood including circular and tubular cross sections because the main densification mechanism is based on gas pressure that is equally exerted in the entire surface, rather than localized mechanical compression, which can only be effective with rectangular cross sections. In the first stage of the two-step proposed method, the compressive strength of the anatomical wood structure is reduced by delignification, and, in the second, a full densification is achieved by hot isostatic pressing under argon atmosphere. Three tropical hardwood species with distinct anatomical characteristics and properties were used to test the method. The HIP-densified wood’s microstructural, chemical, physical, and mechanical properties were assessed. Apart from the high densification values and volume preservation, the results indicate that proposed method was effective for all the tested species, showing homogenous density patterns, stable densification without noticeable shape recovery, and enhanced mechanical properties. Future research should test the HIP method in softwoods and consider the ring orientation in order to enhance the control of the densified geometry.

## Introduction

Two-step densification is a process that improves the mechanical properties of wood by first reducing the cell wall strength by physical or chemical methods (softening of the anatomical structure) and subsequently applying mechanical compression^1,2^. Softening facilitates the wood’s densification^3^ and avoids problems such as shape recovery in densified wood^4,5^. Mechanical densification of wood typically comprises the application of one of the following processing methods: viscoelastic thermal compression (VTC), thermo-mechanical (TM), thermo-hydro (TH), or thermo-hydro-mechanical (THM). All these methods commonly include a densification process of at least two steps, as they firstly soften the structural components of wood with heat or moisture, taking advantage of the viscoelastic properties of the wood, before subsequently applying mechanical compression^6^. However, a drawback these methods share is that the densified wood tends to swell back (shape recovery) when it is subjected to moisture after the process has been completed. Therefore more than two steps are normally necessary to reduce shape recovery, such as additional heating or cooling treatment after pressing, which increases the cost and time of the processing^7,8^.

Multiple physical and mechanical properties improve with densification^9^, some of them showing great increases depending on the compression system used, the wood species, and the processing parameters. Some of the most highly-sought improvements include higher density and dimensional stability, stronger modulus of rupture and modulus of elasticity, and increased compression perpendicular to the grain strength^10,11^. Despite such improvements, the aforementioned densification processes show some disadvantages or limitations in addition to swelling back, such as limited increases in density as well as partial and non-homogeneous densification. For example, it is typically acknowledged that wood cell wall density amounts to 1.5 g/cm^3^^12^; however, THM processing, despite having been shown to improve wood’s density and mechanical strength^13^, only achieves partial densification up to 1.29 g/cm^3^^14^, while the density achieved via TM processing has been reported as only 1.10 g/cm^15^. These typical mechanical compression methods show limitations in removing the empty lumens of the wood in the weakest regions of the structure, and may cause micro-fractures in the cell wall of vessels and fibers^5,16^. Regarding VTC densification, this method has been capable of reaching densities up to 1.4 g/cm^3^^1^, however, it does not completely remove wood porosity and it is limited to the processing of thin wood laminates^1,17,18^. Besides, the VTC method yields non-uniform density patterns^17^. Another limitation of the previously-mentioned mechanical densification methods is that, although wood can be densified in all different directions (radial, tangential, or longitudinal), these techniques reach top densification values only when applied in the radial direction^11,19^. For these reasons, wood treated with any of these methods has limitations in its use.

In this context, new wood densification methods have been researched in recent years with a view to reducing the aforementioned drawbacks, achieving full densification, and further improving wood properties. Equiaxial or isostatic densification may be an alternative method for achieving a more efficient densification. One equiaxial densification technique, high-pressure (HP) treatment, densifies wood in the absence of heat treatment^19^ by applying hydrostatic pressure to vacuum-sealed wood in a polymer bag inside a chamber filled with pure water^20,21^. HP densification can process low-density softwood in a brief time while preserving a significant volume of the treated wood because the material is only pressed in one direction. However, densification with this method is limited up to 1.0 g/cm^3^, which represents a relatively low increase in mechanical properties^21,22^. On the other hand, semi-isostatic compression technology—so-called since compression is not perfectly isostatic-, applies pressure to wood laying on a rigid surface through a flexible oil-filled rubber diaphragm^23^. This rapid, non-thermal processing technique has demonstrated increases in wood density up to approximately 1.0 g/cm^3^^24,25^. Even higher increases have been reported, but the density of the modified wood decreases because of the compression set recovery effect^25^. This technique is only used to process low-density softwood, and it has a significant disadvantage in that does not reduce compression set recovery unless additional treatments are included. A study by Boonstra and Blomberg^26^ reported a reduction of the shape recovery effect in densified wood by including a heat treatment process before the semi-isostatic compression. More recently, the study by Song et al.^2^ proposed a new two-step densification process, which densified both softwood and hardwood up to a density of 1.3 g/cm^3^, significantly improving their mechanical properties, inhibiting the shape recovery effect, and providing excellent resistance to wet conditions without the need to include a post-treatment phase. As a first step, they softened the anatomical structure of the wood by chemical lignin removal (delignification), a process that has been extensively researched in combination with other wood modification treatments^27–30^. The aim of the lignin removal was to expose the cellulose chains in order to subsequently modify the material^28,31^. Although this process is typically focused on the removal of the lignin, it typically leads to also some hemicellulose removal, therefore may be more accurate to refer it as lignin/hemicellulose removal. The disadvantage of the densification process of the aforementioned study was that it still was unable to achieve full densification by vanishing porosity (density of 1.3 g/cm^3^), and also that a significant volume loss of about 79% CR in the radial direction was obtained due to uniaxial densification being found^32^. Finally, it also required a time-consuming process. Other recent works on this densified wood development process achieved similar results^6,10^.

**Figure 1:**
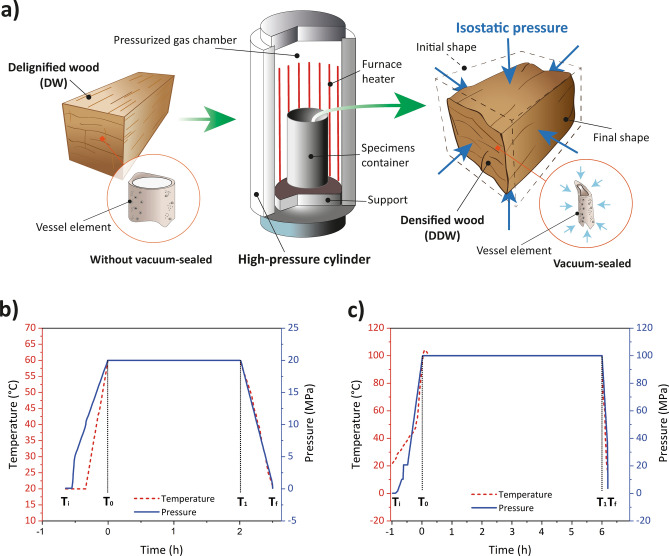
Graphical illustration and parameters of the densification process. (**a**) Illustration of the densification process stages by chemical pretreatment and subsequent hot isostatic pressing. (**b**–**c**) Schematic diagram of the HIP process parameters: preheating (T_i_–T_0_), compression (T_0_–T_1_), and release (T_1_–T_F_). (**b**) Condition 1 (C1) simultaneously applies a pressure of 2900 psi (20 MPa) and a temperature of 60 °C for 2 h of treatment. (**c**) Condition 2 (C2) simultaneously applies a pressure of 14,500 psi (100 MPa) at 100 °C for 6 h of treatment.

In this article, a new two-step densification method consisting of delignification followed by hot isostatic pressing (HIP) is proposed. HIP has been traditionally used for the densification of metals and ceramics by simultaneously heating and applying high isostatic pressure^33^, customarily using argon as the compression gas^34^, which allows for equiaxial pressing^35^. This method has the advantage of allowing equal pressure to be exerted on all surfaces while having control of processing temperature and pressure, as illustrated schematically in Fig. 1a. In this research we explored the applicability of the HIP technique after partial lignin/hemicellulose removal in the two-step process in order to achieve high-performance fully-densified wood with enhanced properties. The effect of isostatic compression on the densified wood's physical/mechanical properties and post-mechanical fixation is evaluated in three hardwood species using compression ratio (CR), water absorption (WA) capacity, SEM imaging, and FTIR spectroscopy.

## Results and discussion

### Effect of the HIP on the microstructure of wood

**Figure 2:**
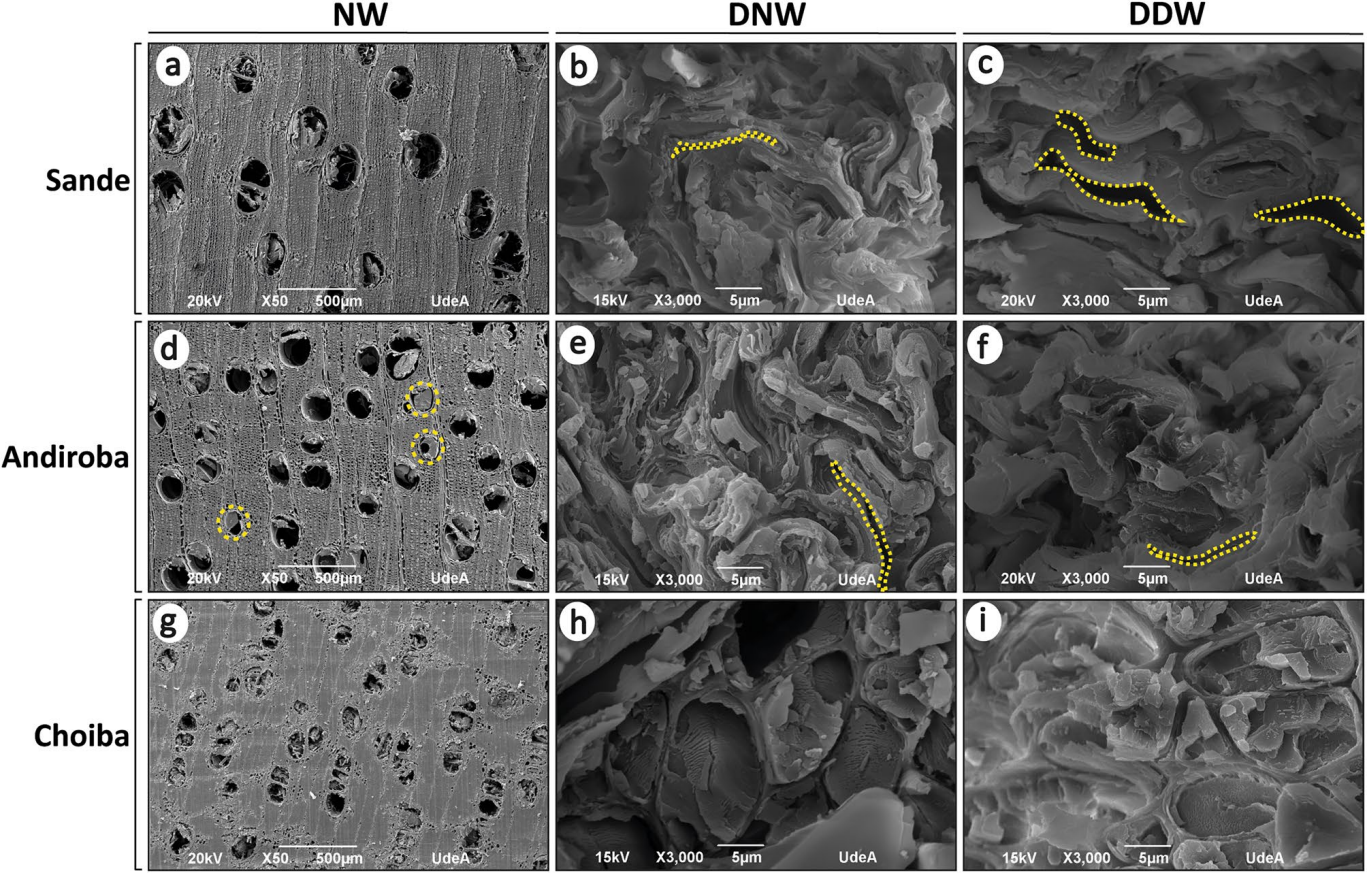
Scanning electron images showing the cross-sections of Sande, Andiroba, and Choiba wood treated by hot isostatic pressure. *NW* natural wood, *DNW* densified natural wood, *DDW* densified-delignified wood. Heartwood vessels are highlighted in yellow.

Scanning electron images showing the cross-sections of Sande, Andiroba, and Choiba wood following a HIP process are displayed in Fig. 2. To establish a clear baseline, it was decided to study the effect of wood delignification on the HIP process. To this end, natural wood (NW) (this condition refers to wood that was neither densified nor delignified), densified natural wood (DNW) (this condition refers to natural wood that was densified without being previously delignified) and densified-delignified wood (DDW) (this condition refers to wood that was previously delignified and then densified) were analyzed. For NW (Fig. 2a,d,g), the surface and internal porosity of the three specimens were comprised of a structure of diffuse-porous vessels, visible to the naked eye. Andiroba wood showed gums in heartwood vessels^36^, see the yellow circle of dashed lines in Fig. 2d. The anatomical characteristics of the three non-densified specimens were consistent with the results of previous observations for each wood species^37^. The fibers of Sande wood consisted of non-septate, thin- to thick-walled fibers with a large lumen. The fibers in the Andiroba wood consisted of septate, thin- to thick-walled fibers with a broad lumen. In contrast, Choiba wood fibers consisted of non-septate, very thick-walled fibers with a small lumen. In Fig. 2b,e,h, corresponding to the DNW following the HIP process, it was observed that the anatomical wood structure had an obvious reduction in porosity. This effect was similar for the DDW (Fig. 2c,f,i). However, different degrees of fiber lumen reduction were obtained from the Sande specimen (Fig. 2b,c). Although a more significant effect on fiber lumen decrease is observed in DNW than in DDW, this behavior is not generalized in the DDW structure and may be attributed, among other factors, to the difference between the lignin contents of DNW and DDW. In addition, in culture cells without cell-wall has been observed that upon equiaxial compression device for mechanical manipulation the cells decrease in cell cross-sectional area, however, increases in cell layer height^38^. Thus, there may be a possibility that heartwood vessels-DDW flexed at the beginning of compression since heartwood vessels from DDW have less lignin than DNW. Furthermore, it is known that the direction of the applied force affects cellular orientation. According to the morphological results, the densification process was effective for both Andiroba and Sande, and the densification was sensitive to previous delignification processes. This is consistent with the fact that wood deformation in compression depends on the specimens used^25^, as the anatomical characteristics of the species influence delignification and subsequently compression. For example, the HIP process was less effective for Choiba than for the other species since the thick-walled fibers, being more difficult to compress, did not suffer significant alterations (Fig. 2h,i), and so a smaller reduction was achieved in the lumens of the anatomical structure of this species. In the SEM images of the DNW and DDW specimens, a marked reduction of the span volume of the cells can be observed, as well as a deformation of the cell walls of the densified wood specimens in the compression direction (in this case equiaxial). Other studies have reported similar behavior^39^. Both the vessel structure and fibers deformed irregularly. According to a previous report, fibers under isostatic compression acquire an irregular shape^25^. Furthermore, the cell walls of DNW and DDW were not damaged and maintained their original integrity, indicating that most of the cell walls of the inner layers were softened by chemical and thermal pretreatment during the densification process^40^. In that sense, the HIP process preserves one of the most important aspects of VTC densification since it modifies the wood in the absence of microfractures. This is considered an important factor in improving densified wood's physical and mechanical properties^17^ because the integrity of the anatomical structure is preserved. In addition, it also demonstrates the importance of the partial removal of the basic chemical components of wood before the densification process.

### FTIR analysis

**Figure 3:**
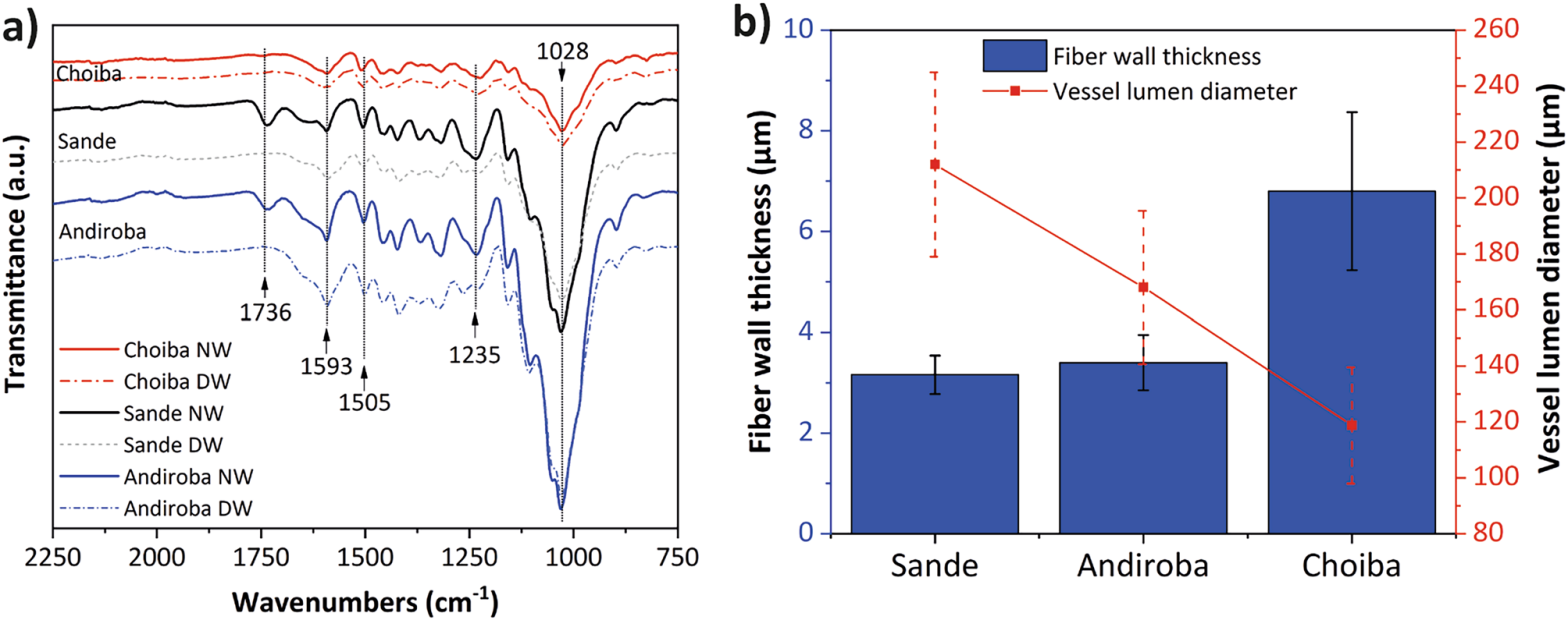
Variations in chemical composition and anatomical aspects of the specimens studied. (**a**) FT-IR spectra of various wood specimens after alkaline chemical treatment (*NW* natural wood, *DW* delignified wood). (**b**) Some anatomical differences of the specimens studied.

Lignin and hemicellulose were partially removed using a sodium hydroxide treatment before the densification process. This delignification procedure reduces cell wall strength and expands the lumens, improving both the interconnections between lumens^30^ and the vacuum performance before the HIP process. By comparison the effect of lumen expansion in densified wood is shown in Fig. 2b,c. Figure 3a shows the FT-IR spectra of wood specimens, which are compared for NW and delignified wood (DW). According to these results, the absorption of the bands located at 1736 and 1235 cm^−1^ for carbonyl stretching and C–O stretching of the guaiacyl unit of lignin^41^, is significantly reduced, which demonstrates the removal of lignin and hemicellulose^42,43^ in delignified Sande and Andiroba woods. For Choiba, this behavior was not observed. It should be noted that delignification was performed analogously in all specimens. Compositional analyses determined the relative lignin, hemicellulose, and cellulose in the specimens studied (Supplementary Fig. S1). Lignin and hemicellulose reductions were shown to occur in the thin cell wall wood specimens. Although the fiber wall thickness is similar between Sande and Andiroba (Fig. 3b), the lignin removal in Andiroba was higher than expected (Supplementary Fig. S2), even when Andiroba has a smaller vessel lumen diameter than Sande species. These differences in the effect of the delignification process between Sande and Andiroba can be explained by variations in the anatomical characteristics of the specimens, as well as by the reactions occurring during delignification, which have been described extensively in previous studies^44^. The decrease in lignin in the Sande specimen due to the delignification process was as expected. Song et al.^2^ demonstrated that a 45% reduction in lignin is ideal for the densification process. The typical absorbance at 1539 and 1506 cm^−1^ for the aromatic skeleton in lignin^42^ showed no significant variation in lignin from the reduction of band intensity after treatment. This showed that the lignin component was partially preserved with delignification. The intensities of the bands at 1028 and 1740 cm^−1^ (Fig. 3a) were observed to be similar in each wood specimen after alkaline treatment, which indicates that the cellulose was not removed^45^. In general, delignification treatment degrades some components of the wood cell wall (lignin/hemicellulose) and, as a consequence, generates pores in the anatomical structure that facilitate wood compression during the densification process^46^.

### Physical properties

**Figure 4:**
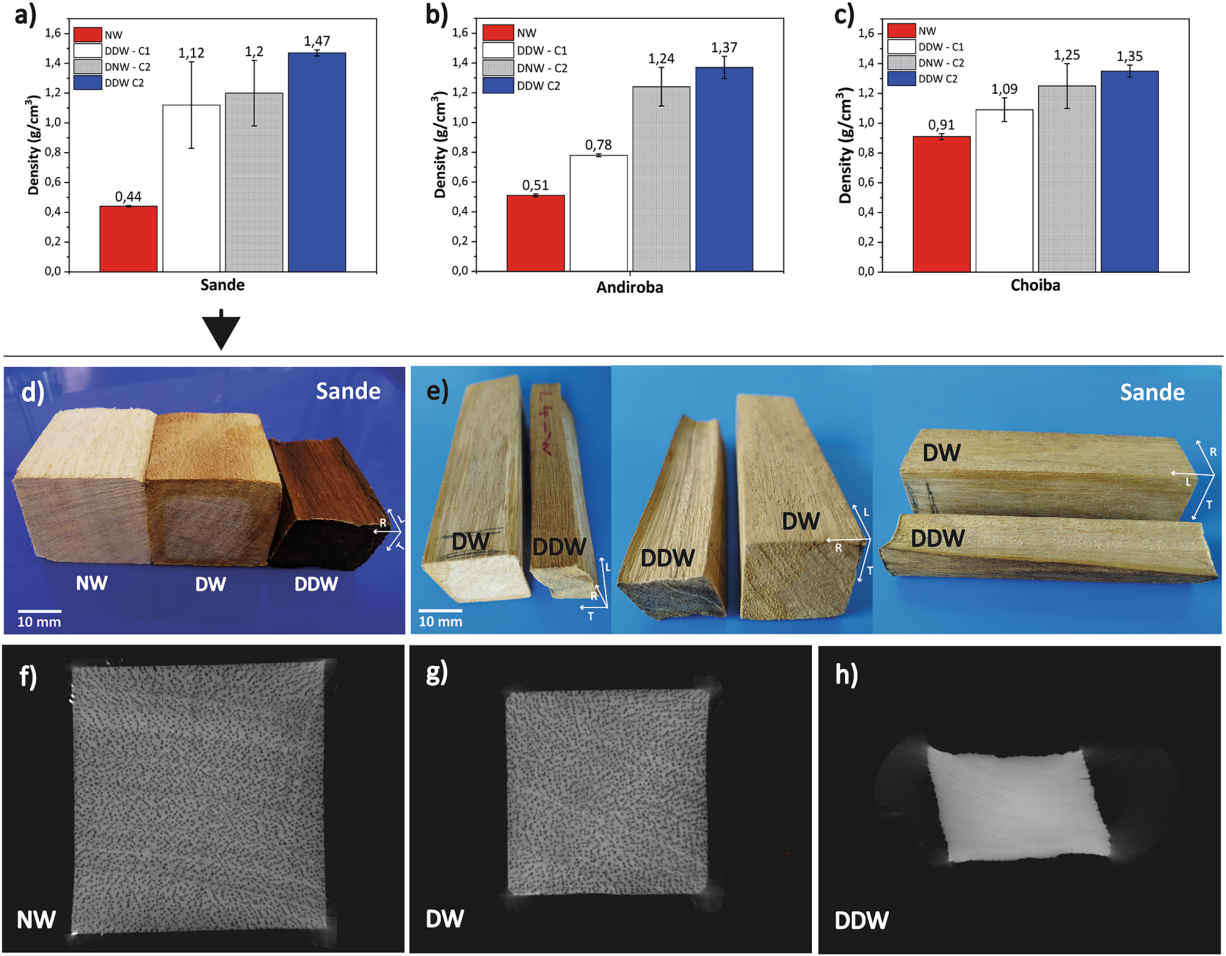
Physical properties and appearances of natural and delignified-densified wood. Density evolution of (**a**) Sande, (**b**) Andiroba, and (**c**) Choiba. *NW* natural wood, *DW* delignified wood, *DNW* densified natural wood, *DDW* densified-delignified wood, *C1* condition 1 for the HIP process, and *C2* condition 2 for the HIP process, (**d**) and (**e**) Physical of Sande following different treatments, (**f**) NW, (**g**) DW and (**h**) DDW optical densities.

The physical properties and appearances of natural and delignified-densified wood are presented in Fig. 4. The densities of NW vary in the range of 0.44–0.91 g/cm^3^. After the HIP process, for any HIP condition (i.e., C1 or C2), and regardless of whether the delignification procedure was performed or not, the density of the hardwoods increased. For HIP process condition C1 (i.e., 20 MPa, 60 °C and 2 h), density increased in all three hardwood species. It is important to note that only DW was used for C1. The greatest increase occurred for the Sande species. The largest increase, about 2.6 times in comparison to natural wood, was for Sande (Fig. 4a), while Andiroba (Fig. 4b) showed an increase of 1.5 times and Choiba (Fig. 4c) 1.2 times. On the other hand, HIP process condition C2 (i.e., 100 MPa, 100 °C, and 6 h) led to a higher density increase. In C2, both NW and DW were densified. Comparing DNW with NW, the density showed increases of 2.7, 2.44, and 1.4 times for Sande, Andiroba, and Choiba, respectively. However, for DDW, even larger increases of 3.3 times 2.7, and 1.5 times were respectively obtained. Considering these effects on density, we believe that, among other aspects, thus, wood density increases with increases in HIP process parameters (pressure, temperature, and time); however, because only two sets of parameters were tested in this novel investigation, further processing parameters should be analyzed in the future to find the optimal processing parameters for HIP densification of each wooden species. Furthermore, it is noted that the increase in density was more positively affected in those specimens where delignification pretreatment was previously performed. Moreover, the greatest increase in density occurred in those specimens where a greater amount of lignin was removed, while the smallest increase in density was for Choiba. The last of these results may be due to the small fiber lumen and wall thickness hindering cell wall compression^25^. In addition to the foregoing, the vacuum state of the fiber and vessel lumens during the HIP process allowed the cell wall of all three specimens to collapse (Supplementary Fig. S3). During this step, the elevated gas pressure reduces the lumens of the anatomical structure by compressing the softened cell wall through both chemically and temperature effects during processing, allowing the cell wall not to fracture during deformation. The increased density of DNW and DDW specimens was mainly attributed to the decrease in cell lumen volume. As shown in Fig. 3b, Sande showed the highest vessel lumen and the lowest fiber thickness, while Choiba showed the inverse, that is, the lowest vessel lumen and the highest fiber thickness. Therefore, after the HIP process, the highest density value was reached in the specimen with the lowest density value, in which the previous delignification process had the most noticeable effect. Sande DDW reached a maximum density value of 1.49 g/cm^3^ (~ 239% higher than that of the Sande NW). It has been reported that the cell wall of this species shows a density value of 1.5 g/cm^3^^12^; consequently, this result indicates that the combined effect of the chemical pretreatment (partial removal of lignin and hemicellulose) alongside the HIP process allows the wood to be densified almost up to its cell wall density, i.e. porosity is almost completely removed. SEM images confirmed that Sande DDW retained a small part of the lumens of the porous structure without completely collapsing (Fig. 2c). This effect was even observed to a lesser extent in Sande DNW (Fig. 2b); however, it had no major effect on density. This conclusion is further reinforced when studying the homogeneity of density using optical density in Sande. While annual rings and other regions of different densities are clearly visible in the measurements of natural wood (Fig. 4f) and delignified wood (Fig. 4g), the optical density of delignified-densified wood (Fig. 4h) reveals a very homogeneous structure where only the annual rings are barely visible. This is evidence that HIP densification gives the modified wood a homogeneous density; especially with higher processing conditions (C2). It also demonstrates the effectiveness of the equiaxial pressure in densifying the wood equally throughout its entire structure.

Table 1 shows natural wood and CR shrinkage values after the HIP densification process. The CR values were derived from the HIP process (C2). Although the nature of the densification process is isostatic (or equiaxial), the compressibility of densified wood occurs mainly in the tangential direction (Fig. 4d,e). In this process, failure is radial and tangential, but selectively over the weaker direction^47^. Compression tended to be greater in the tangential direction, as the rays tended to have a restraining effect on compression in the radial direction. This differs from Blomberg's predominance of compressibility in the radial direction^23^. However, this is the main reason why such a high density was obtained in the Sande species. This predominant increase in density due to compression in the tangential direction has been described elsewhere^39^. In the present case and considering that the HIP process acts on the region that gives less resistance to compression, this effect was because the natural wood under study had a higher incidence of tangential shrinkage. As shown in Fig. 4d, the result of the HIP process was dominated by the compression of the specimen in the tangential direction, followed by of the compression in the radial direction. In contrast, the longitudinal direction remains almost unaltered. This pattern was observed in all three specimens. According to the results, the highest CR was 65% in DDW Sande. Although Choiba NW presented higher shrinkage values, the effects of the HIP process were the opposite, i.e., density values were not improved. This was because Choiba presents an anatomical structure that blocks the chemical removal of lignin and hemicellulose and therefore limits wood densification, as explained earlier. In addition, the results showed that the higher compression ratio in the densified wood in all three specimens is related to delignification, since lower compressibility values are associated with densified specimens that have not been previously delignified. This demonstrates that the role of the delignification process before the HIP process is of vital importance. In other words, in the present work, high wood density values were achieved by simultaneous compression of all faces of the specimens, while preserving a significant volume of the material. This is highly interesting as may suggest different uses or applications for the treated wood since various studies have pointed out that volume loss is one of the problems that emerge during wood compression^13^. Therefore, the HIP process overcomes one of the major disadvantages of traditional densification methods, which is the significant loss of wood volume. Previous studies have reported a CR of approximately 80% in the radial direction of densified wood to obtain a partial increase in density^2,10^. In contrast, our study achieves the theoretical density of the cell wall, guaranteeing a greater useful volume of wood. In this study, Sande DDW and NW under the HIP process (C2) showed an average CR in the radial direction of the wood of approximately 29.2% and 24.4%, respectively. In the tangential direction of the wood, the average CR was approximately 50.2% and 37.7% for DDW and NW, respectively. Meanwhile, Sande DDW under the HIP process (C1) showed an average CR of approximately 18.5% and 40.2% in the radial and tangential directions, respectively. This shows that pretreatment with an alkaline solution influences the higher CR of densified wood^10^. Process parameters, such as pressure and temperature, also played a role in increasing CR. Traditional methods control the CR during densification using metal buffers^5^. In contrast, the HIP process does not control this aspect, and methods of controlling it have not yet been studied. Therefore, we consider that one of the reasons for the greater volume preservation by the HIP process in densified wood is isostatic compression, as the cell wall is compressed at the same time in different directions.

**Table 1:** Shrinkage coefficient, anisotropy natural wood, and a densified wood compression ratio.

Specimen	Shrinkage coefficient (%)				Anisotropy R/T	Compression ratio (CR)	
	Radial	Tangential	Longitudinal	Volumetric		DNW (%)	DDW (%)
Sande	4.43 ± 0.009	6.47 ± 0.001	0.20 ± 0.001	11.10 ± 0.012	1.52 ± 0.39	53.5 ± 0.12	65.3 ± 0.04
Andiroba	3.74 ± 0.003	6.78 ± 0.007	0.18 ± 0.001	10.71 ± 0.007	1.83 ± 0.24	55.2 ± 0.04	56.1 ± 0.04
Choiba	5.30 ± 0.008	7.49 ± 0.008	0.18 ± 0.0004	12.97 ± 0.015	1.44 ± 0.24	20.0 ± 0.10	31.3 ± 0.02

It can be recognized that on the other hand the HIP also generated a distorted (deformed) cross section as illustrated in Fig. 4h because the wood tended to be compressed following the weakest directions, which strongly depends on the ring orientations. This distortion can lead to an additional loss of material when further processing the wood to a regular shape. For instance, in this investigation it was found that the resulting CR of the HIP process was 65%, which is a gain of about 15% respect to previous investigations. However, if further processing to a regular shape, an additional 10% of material would be lost, minimizing the gains of the HIP process. A possible solution to this may be testing differently oriented cross sections to minimize distortion, as well as using densified plies to engineer densified wood laminates. These approaches may maximize the material gains of the HIP densification method.

Another relevant aspect is the color change in some DW specimens and in some DDW specimens compared to the color of NW, an effect evidenced in Fig. 4d. This change in wood coloration is due to two factors: exposure to chemical agents during the delignification process and thermal exposure during the densification process, and the reasons why this color change occurs have been extensively explained elsewhere^10,40,48–50^. During chemical exposure, some functional groups are altered (removal of chromophores from lignin, breaking of chemical bonds, and removal of extractives) while during thermal exposure some water-soluble compounds are altered.

**Figure 5:**
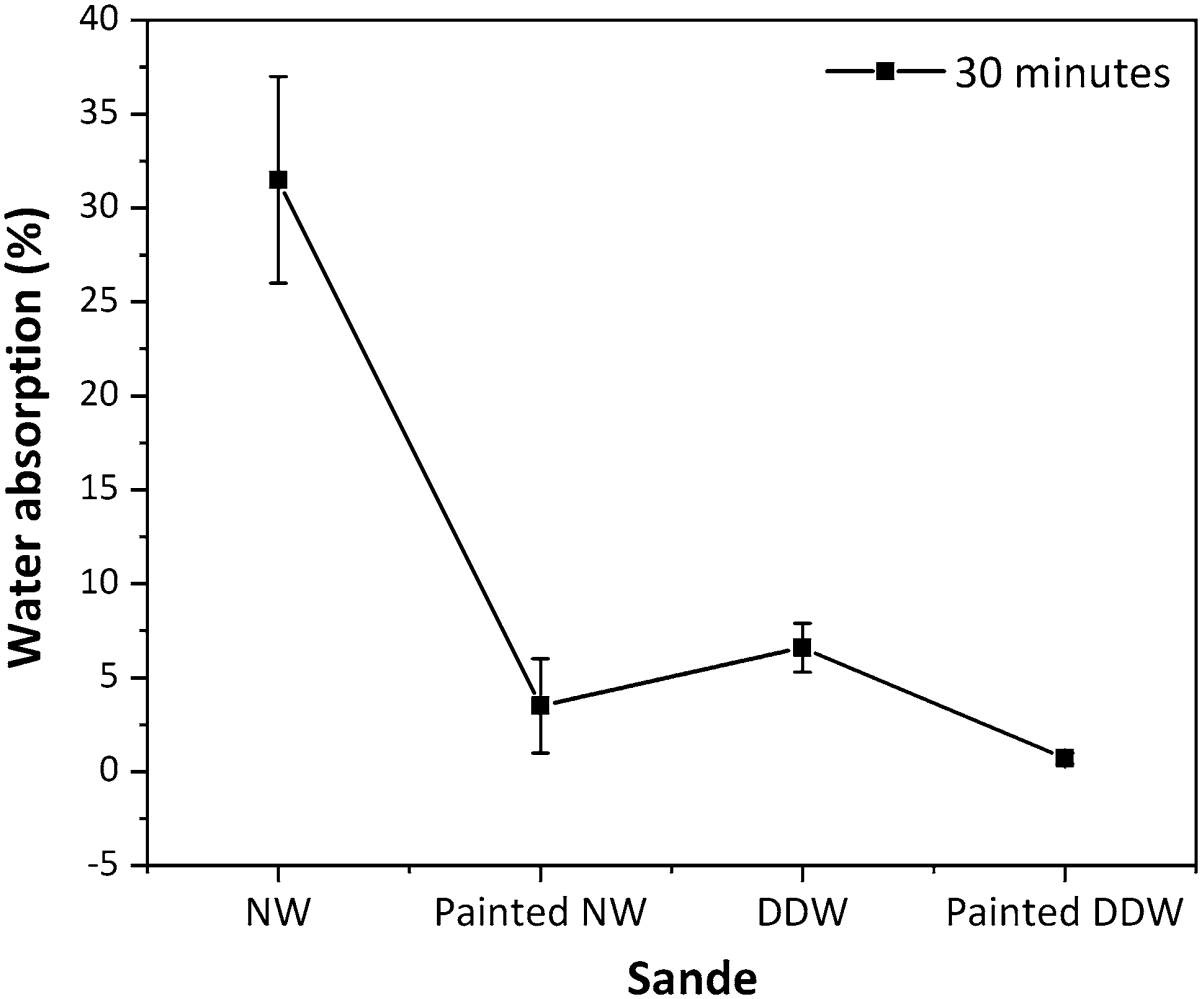
Water absorption (WA) behavior of Sande at different conditions (NW, NW painted, DDW, and DDW painted) following full immersion in water for 30 min.

The volume of the specimens was measured before and immediately after the HIP process to determine the degree of compressive strain. Blomberg J, et al. reported that immediate shape recovery can reach values of 40%^47^. In contrast, after the HIP process, no immediate compressive shape recovery effects were observed in this study. According to previous reports, dimensional stability benefits from the implementation of post-treatments such as thermal modification^5^, and hydrothermal fixation treatment^51^, among others. This investigation did not involve additional treatments to eliminate shape recovery. We inferred that the hot pressing and the gradual release of parameters such as pressure and temperature at the end of the HIP process are among the reasons that explained the adaptation of the densified wood to its new state (compressed). These results are similar to those obtained by Laine et al.^5^, who by applying a thermal modification treatment on densified wood for 6 h at 200 °C significantly eliminated shape recovery. To confirm this inference, in this study, the effects on immediate shape recovery were studied at a temperature of 100 °C for 6 h of HIP process. Furthermore, the wood was not forced into a shape completely foreign to its anatomical structure, as is the case with uniaxial pressing. To further verify the possibility of shape recovery, immersion shape recovery tests were performed on the Sande specimen, since it was the species that showed both a high degree of delignification and proper densification behavior. Figure 5 shows the WA behavior of Sande at different conditions (NW, NW painted, DDW, and DDW painted) following full immersion in water for 30 min. Evidently, WA was higher for NW than for the other conditions. Moreover, there was a higher WA for those specimens that were not painted compared to those that were painted. However, the DDW showed water absorption slightly over (6.6%) that of the painted NW (3.5%). The implication is that the two-step process for wood densification proposed here can fully vanish the wood porosity, preventing WA for 30 min in a manner equivalent to that expected from commercial wood varnishes. This is because, during the HIP process, there is a reduction in the number of cell lumen of the treated wood, as explained previously. The closure of fiber and vessel cells showed a significant effect on TS, since the moisture absorption method presented a difference of 144% between NW and DDW, the swelling value being higher in NW. For TS, the DI water immersion method showed the opposite effect, with lower TS in NW than in DDW. Compared to painted DDW, a lower swelling value was observed in painted DDW than in NW. We note that this improvement in the dimensional stability of densified wood is similar to that found in the previous study of Song et al.^2^, who determined that wood densified in a two-step process and with surface coating with paint could withstand humid environments without alterations to their dimensional stability. By comparison, wood treated with our two-step densification process resists water immersion, which enables the developed material to improve its strength even in geographical locations with high rainfall. This is because the process promotes the hygroscopic dimensional stability of the material after the reduction of the number of hydrophilic groups by the degradation of hemicellulose^40^. After the removal of hemicellulose and lignin during delignification, the ability of wood to adsorb water molecules is reduced^52^, and this is further enhanced by coating the closed pores. In fact, this effect could be strengthened by the degradation of hemicellulose by the high temperature during the densification process^53^. We consider that the incorporation of heat during densification significantly decreased the sensitivity of wood to moisture. Although this is a positive effect, dimensional stability could be standardized by a detailed study of the thermal variable of the process without including an additional step. It has been reported that the thermal phase provides better dimensional stability to the densified wood^54^, but it also has significant adverse effects on the physical and mechanical properties of the wood due to the degradation of its chemical components^55^. Finally, it is important to mention that total immersion tests were also performed for 24 h, nevertheless, the results of these tests were not statistically conclusive and, so they were not included in the present work.

### Effect of HIP on mechanical properties

**Figure 6:**
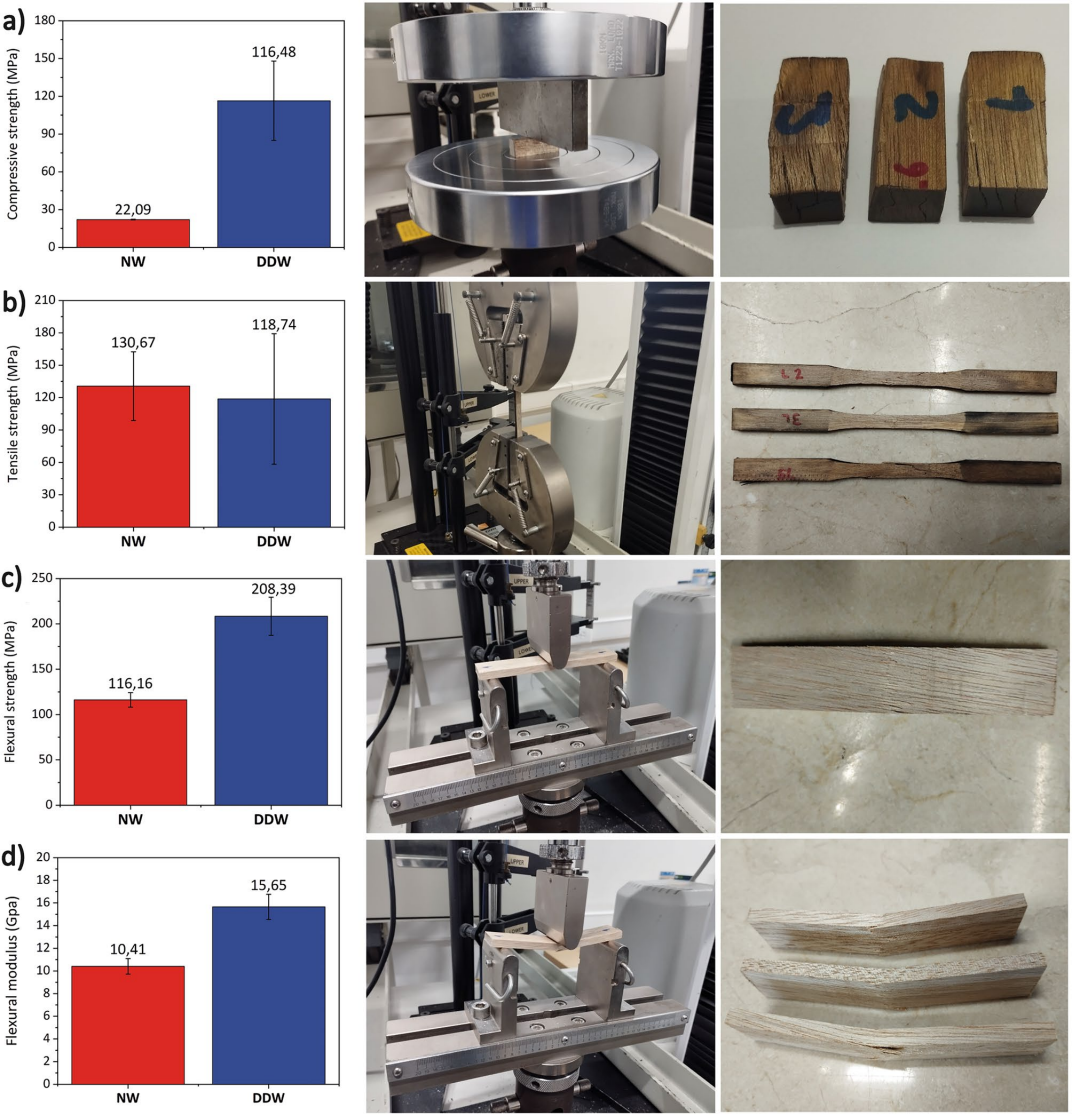
Sande mechanical properties following the two-step densification process. (**a**) perpendicular-to-the-grain compressive strength, (**b**) tensile strength, (**c**) flexural strength, and (**d**) bending modulus.

Figure 6 shows Sande mechanical properties following the two-step densification process. The perpendicular compressive strength of DDW was ~ 5.2 times greater than that of NW, while its tensile strength was shown not to be affected by the two-step densification process, as values derived from mechanical tests yielded no significant statistical differences between DDW and NW. In addition, wood bending tests show that the strength and flexural modulus increased ~ 1.8 and ~ 1.5 times, respectively compared to that of NW. The lack of improvement in the tensile strength of densified wood could be explained by the fact that, by its nature, wood exhibits brittle behavior when subjected to tensile forces. Therefore, after densifying the wood and having partially removed the lignin, it is possible that the cellulose fibers could not improve this behavior; on the contrary, it seems that after the densification process, the fibers fractured a little earlier than those of the natural wood. This means that the densified wood did not increase in stiffness during tensile stresses, yet its stiffness did increase during compressive stresses. This explains the gain in both strength and stiffness of the wood during flexural tests, since, in general terms, the flexural strength of materials is governed by both compressive strength and tensile strength. This improvement in flexural strength could also indicate that the portion of neutral fiber during bending is no longer located right in the middle of the specimen, but is rather displaced a little higher, i.e., the area of the specimen subjected to compressive stress is much smaller compared to the area subjected to tensile stress. The results of the mechanical tests previously discussed are promising since they suggest potential uses or applications of the wood treated by the two-step densification process proposed in this work. For example, wood beams used in numerous structures are designed considering the main parameter the bending deformation. Therefore, the densified wood obtained here could be used to reinforce laminated timber beams. This reinforcement would consist of a densified wood plate located on the outermost part of the laminated beam, thus increasing the flexural strength of the element in question. Another important use could be derived from the exceptional gain in compressive strength. Normally, the mechanical connections between structural elements are the weakest and least rigid points. These results suggest that the replacement of steel elements (particularly plates) in timber structures could be carried out using densified wood plates, as the embedding strength and fasteners’ stiffness closely relates to the wood density^56^. Another structural application could be manufacturing bearing plates, in which both perpendicular-to-the-grain compression strength and stiffness are essential.

## Conclusion

In conclusion, in this work the potential of a two-step HIP wood densification method was investigated in three tropical hardwoods. The method has been shown to be very effective for all three investigated species, with one of them reaching full densification up to 1.47 g/cm^3^, which is most complete densification reported for a hardwood species. Furthermore, the method has demonstrated clear advantages through its preservation of the virgin volume to a large extent, thanks to isostatic pressing. The obtained material also showed advantages in compared to previous densification methods regarding dimensional stability, avoidance of elastic shape recovery and exceedingly small water absorption. SEM analysis showed that specimens with a homogeneous anatomical structure, non-thick cell wall, and no marked defects in their anatomical structure are preferable for the efficient removal of basic chemical compounds from the wood. In addition, SEM also revealed that densified wood made from delignified wood had some lumens that were not completely closed, indicating that natural wood can be further compressed under this method. However, the density achieved in the Sande specimen significantly equals the cell wall density. A high degree of homogeneity is also presented in the densified wood. The material also showed enhanced mechanical properties, with an increase of about 5 times in compression perpendicular to grain, 2 times more flexural strength and 50% more flexural stiffness. Tensile strength was not appreciably increased, which may be attributable to the large brittleness of this property. The above results suggest that the use of HIP densification in wooden materials has great potential, overcoming several limitations of previous wood densification methods. New functionalities and advanced wood-based materials may be investigated given the unique features achieved with these densification properties. The effectivity and relative physical and mechanical gains of the proposed method may be even greater for other lighter species, since all those investigated in this research were medium-density tropical hardwoods. Future research should focus on testing the method in softwoods. Furthermore, it should be investigated if the resulting shape can be controlled by considering ring orientation, because the material utilization would be enhanced by assuring that the resulting shape fits with the target geometry. In principle, this method should be capable of densifying any shape, not only rectangular cross sections, which is an important advantage that also should be assessed in future investigations.

## Materials and methods

### Materials

Three tropical hardwood species namely: Sande (*Brosimum utile*), Andiroba (*Carapa guianensis*), and Choiba (*Dipteryx oleifera*) were considered in this research. The specimens were obtained from the trunks of different trees and measured 35 × 35 × 170 mm (radial × tangential × longitudinal). Before processing, all specimens were kiln-dried up to constant weight at 103 °C for 24 h. Sodium hydroxide (≥ 99.0%) and sodium sulfite (≥ 97%) were purchased from Merck (Canada). Deionized (DI) water was used as the solvent to process the wood. All chemicals were used as received.

### Two-step process toward densified wood

The proposed method consists of a two-step processing. This entails a chemical pretreatment, which leads to partial lignin/hemicellulose removal, followed by a HIP process, in which wood becomes densified. In the first step, delignification is achieved by immersing the specimens in a boiling mixed aqueous solution of 2.5 M, NaOH and 0.4 M, Na_2_SO_3_ at 90 °C for 7 h, followed by immersion in boiling distilled water several times to remove the excess chemical reagents. Previous studies present more details of this process^2,28^. Next, the delignified wood (DW) is oven-dried at 50 °C to 12% moisture content and prepared with dimensions of 30 × 30 × 150 mm (radial × tangential × longitudinal). That is, the ends of the DW were removed before densification process (Supplementary Fig. S4). The DW specimens are then vacuum-sealed at room temperature and covered in a high-temperature-resistant, flexible aluminum bag. Finally, the HIP process is applied. In this research, the HIP system consisted of an HP830 machine, American Isostatic Presses, Inc., Ohio, USA, equipped with a crucible to contain the specimens. The HIP process used a high-purity (99.999%) argon atmosphere. The schematic illustration of the preparation procedure of the HIP densified process is presented in Fig. 1a. The wood specimens were compressed by the HIP in an equiaxial direction. During the HIP process, the specimens were compressed using two different control parameter conditions (temperature, pressure, and time). The first condition (C1) consisted of simultaneously applying a pressure of 2900 psi (20 MPa) and a temperature of 60 °C for 2 h of treatment (Fig. 1b). The second condition (C2) used a pressure of 14,500 psi (100 MPa) at 100 °C for 6 h of treatment (Fig. 1c). Subsequently, the pressure was gradually released over approximately 30 min to counteract shape recovery effects in both conditions. Figure 1,c illustrate the applied pressure and temperature as a function of time during the HIP process. Three replicates were performed for each group of specimens and pressure condition.

### Measurements and characterization

A scanning electron microscope (SEM, JEOL JSM-6490LV) operating at 15 and 20 kV and 85 amperes was employed to evaluate the morphology and microstructure of the cross-section of the three densified wood specimens. The densified specimens were sectioned by the cryofracture technique. The observed characteristics were measured by SEM analysis and using the image analysis ImageJ software^57^. The Fourier transform infrared (FTIR) (Spectrum Two, PerkinElmer, MA, USA), using a device equipped with an attenuated total reflectance (ATR) module, was performed in range from 4000 to 450 cm^−1^ at a resolution of 4.0 cm^−1^ and taking the average of 24 scans for each specimen. The natural wood (NW) and densified wood were prepared as thin sections, with thicknesses lower than 1 mm. One measurement was performed per specimen. The spectra were recorded as transmittance versus wavenumber to find the effect of the HIP process in the specimen. The number of chemical components (carbohydrates and lignin) measured are not presented in this study.

Moisture content was measured before and after each process step by oven drying at 50 °C to constant weight. The bulk density of the three specimens was determined before and after the HIP process. Density was evaluated by directly measuring their volume with a Mitutoyo micrometer (precision of 0.001 mm) and their weight with a RAD WAG-AS 310.R2 electronic microbalance (accuracy of ± 0.1 µg). In addition, to visualize the homogeneity of the densification process, the optical density of the specimens was measured using microcomputed tomography with the SkyScan 1278 at the University of Chile, Santiago, Chile. The shrinkage ratio of the three specimens was evaluated before and after the HIP process. The compression ratio of the densified wood volume was measured from the ratio of the initial and final volume of the specimens in dry conditions before and immediately after densification, respectively. For dimensional stability (thickness swelling—TS), pre-cut specimens of the rectangular cross-section of specimens with dimensions of approximately 15 × 15 × 30 mm (radial × tangential × longitudinal) were fully immersed in DI water for 30 min at room temperature. Before the full immersion test, specimens were painted on all sides using commercial varnish for exteriors. The 30-day moisture absorption method for the species was also used. For this method, specimens were placed in a stable environment at 20 °C and 65% RH for 30 days. The dimensions and weight of the pieces before and after immersion and humidity conditions were recorded. The weight gain (g g^−1^), denoted as the mass of water (g) adsorbed per unit mass of dry densified wood (g), was used to evaluate WA capacity. The mechanical properties of the NW and densified wood were evaluated using an Instron testing machine with a capacity of 10 and 150 kN. Three-point static bending tests were used to determine the bending properties using the modified standard procedure outlined in ASTM D143-21^58^ with a specimen dimension of about 5 × 20 × 100 mm (radial × tangential × longitudinal). The compressive strength was measured according to the ASTM D143-21^58^ standard, in which specimens for compressive testing should have dimensions of 10 × 10 × 30 mm (radial × tangential × longitudinal). Tensile properties were determined in specimens that were cut to the dimensions of 3 × 10 × 140 mm (radial × tangential × longitudinal). The specimens were loaded at a constant loading rate until they failed. Before mechanical properties, specimens were conditioned at 65% RH and 20 °C until a constant mass was obtained. The tests were conducted at 20 °C and 55% relative humidity. At least three replicates were measured to calculate the mean value of the measurements. Origin Pro 2018 was employed to analyze all data and plot figures.

### Research involving plants

The authors of this study declare that the collection and use of any plant material in this study was carried out in accordance with the guidelines and regulations of the national government of Colombia.

### Supplementary Information

Supplementary Figures.

## Data Availability

All data generated or analysed during this study are included in this published article.
