# Supplementary material for: Two-step hot isostatic pressing densification achieved non-porous fully-densified wood with enhanced physical and mechanical properties
Source: Sci Rep. 2023 Aug 31;13:14324. doi: 10.1038/s41598-023-41342-8 (PMC10471585; doi:10.1038/s41598-023-41342-8)
Supplement: Supplementary file 1 — Supplementary Figures. [file 41598_2023_41342_MOESM1_ESM.docx]

**Supplementary Information**

**Two-step hot isostatic pressing densification achieved non-porous fully-densified wood with enhanced physical and mechanical properties**

J.C. Maturana^1,5^*, P. Guindos^2^, J. Lagos^2^, C. Arroyave^3^, F. Echeverría^4^, and E. Correa^1^*

^1^ Grupo de Investigación Materiales con Impacto – MAT&MPAC, Facultad de Ingenierías, Universidad de Medellín UdeMedellín, Carrera 87 No. 30 – 65, 050026 Medellín, Colombia

^2^ Centro Nacional de Excelencia para la Industria de la Madera (CENAMAD), School of Engineering, Pontificia Universidad Católica de Chile, Vicuña Mackenna 4860, Santiago, Chile

^3^ Grupo de Investigaciones y Mediciones Ambientales – GEMA, Department of Environmental Engineering, Universidad de Medellín UdeMedellín, Carrera 87 No. 30 – 65, 050026 Medellín, Colombia

^4^ Centro de Investigación, Innovación y Desarrollo de Materiales – CIDEMAT, Facultad de Ingeniería, Universidad de Antioquia UdeA, Calle 70 No. 52-21, Medellín, Colombia

^5^ Grupo de Investigación Valoración y Aprovechamiento de la Biodiversidad - VALORABIO, Universidad Tecnológica del Chocó UTCH, Carrera 22 No. 18B – 10, Quibdó, Colombia

* Corresponding authors: J.C. Maturana (jmaturana696@soyudemedellin.edu.co); E. Correa (escorrea@udemedellin.edu.co).

**
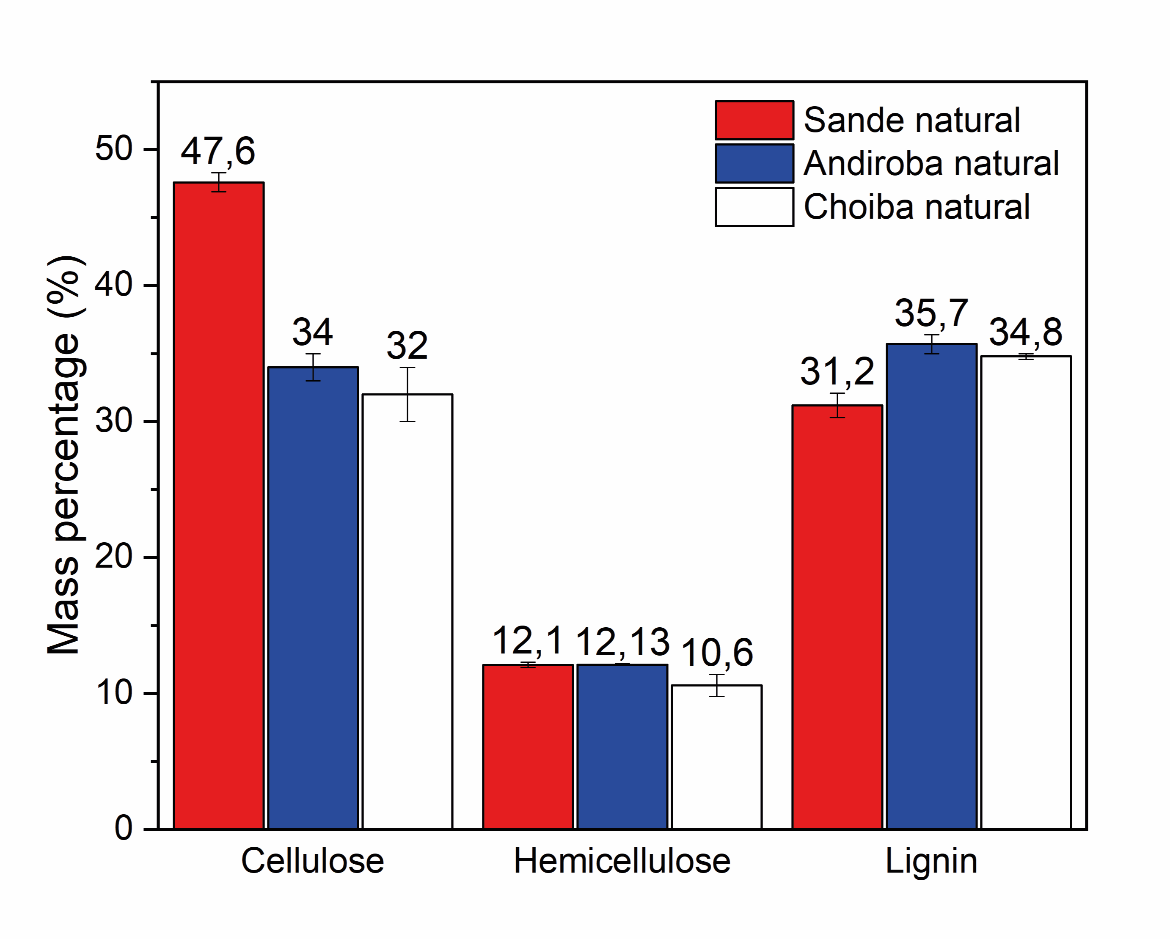
**

**Supplementary Figure S1.** Relative cellulose, hemicellulose, and lignin content in the natural wood of the specimens studied.

**
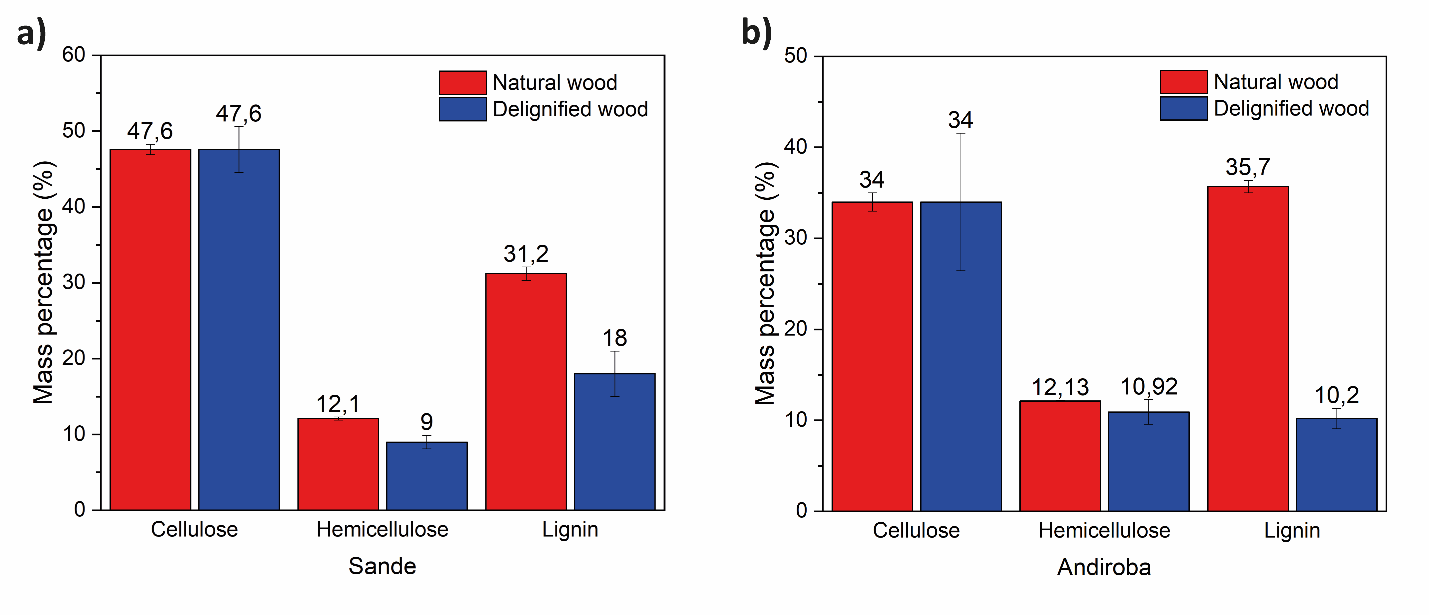
**

**Supplementary Figure S2.** Comparison of relative cellulose, hemicelluloses, and lignin content in delignified wood. a) Delignified Sande. b) Delignified Andiroba.

**
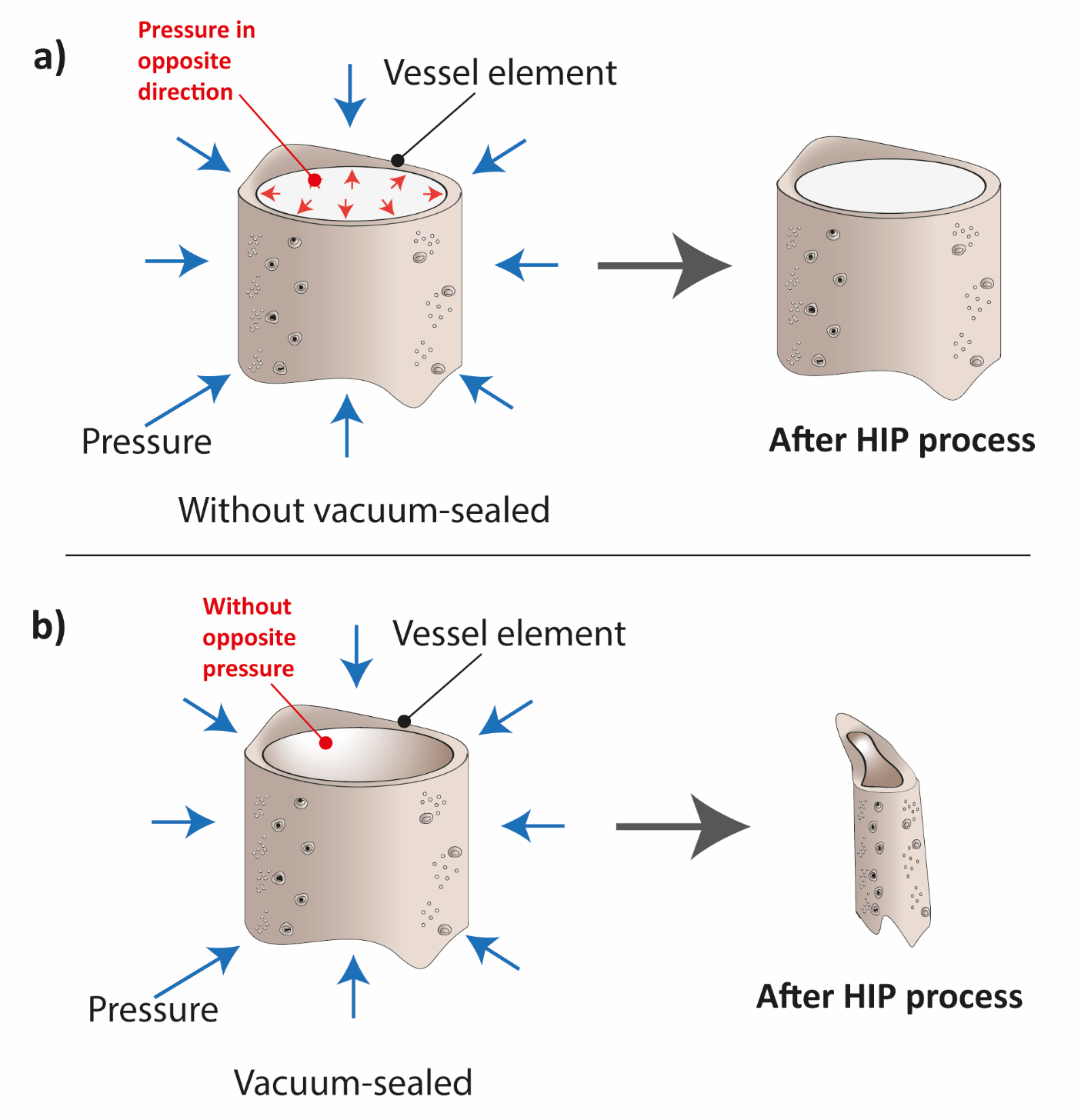
**

**Supplementary Figure S3.** Graphical illustration of the anatomical effect of the HIP densification process. (a) Nullification of the densification pressure due to the presence of fluids inside the cell lumens that provide an opposing pressure to the process. (b) Reduction of the cell lumen generated by the densification pressure in the absence of an opposing pressure from inside the cells.


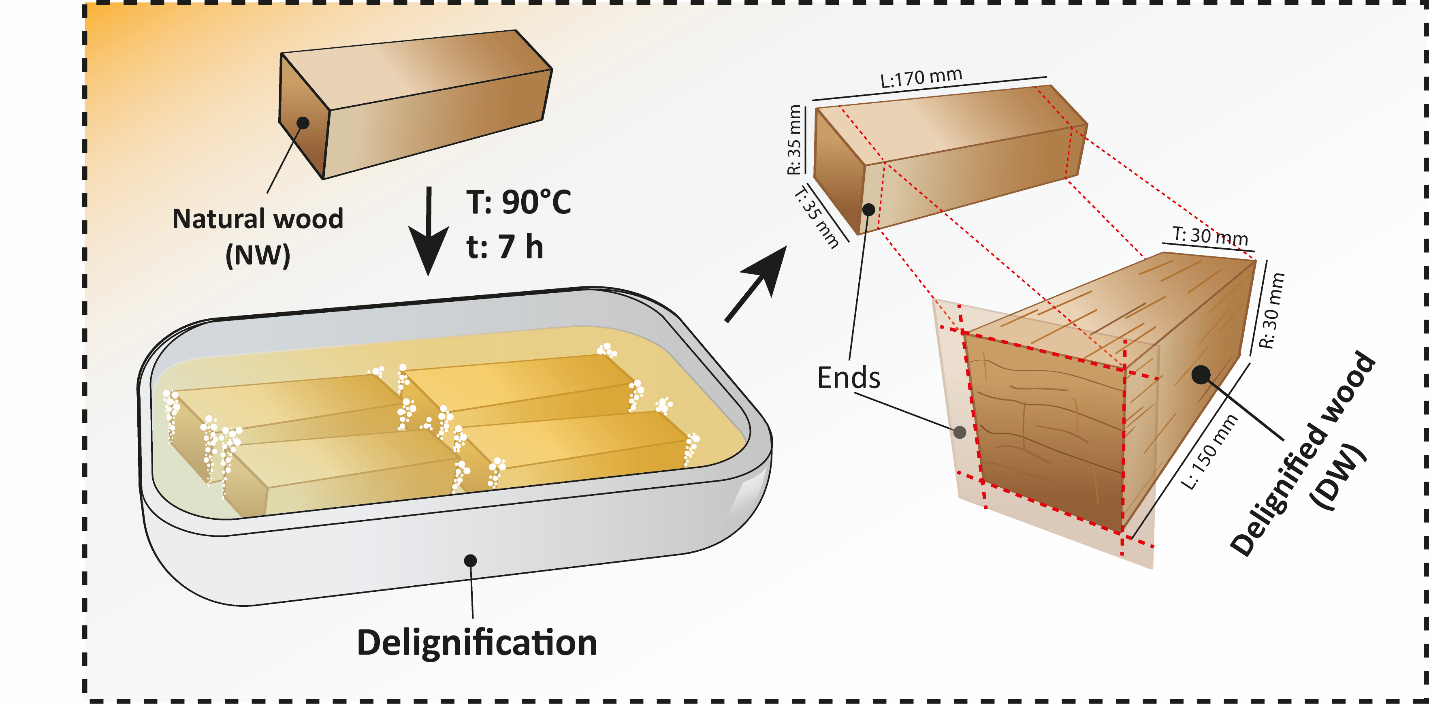


**Supplementary Figure S4.** Graphical illustration of delignified wood preparation.
